# Refined detection of CD34⁺CD38⁻CD45RA⁺ leukemic stem cells using a single-tube flow cytometry assay and its strong association with measurable residual disease in acute myeloid leukemia: a retrospective cohort study

**DOI:** 10.1186/s13287-026-05038-w

**Published:** 2026-05-02

**Authors:** Amir Abbas Navidinia, Shahrbanoo Rostami, Najibe Karami, Mohammadreza Shemshadinia, Habibeh Sabri Patekhor, Akram Hajaliaskari, Saeed Mohammadi, Tahereh Rostami, Maryam Barkhordar, Mohammad Vaezi, Ghasem Janbabaei, Bahram Chahardouli

**Affiliations:** 1 Hematologic Malignancies Research Center, Research Institute for Oncology, Hematology and Cell Therapy, Shariati Hospital, Tehran University of Medical Sciences, Tehran, Iran; 2 Cell Therapy and Hematopoietic Stem Cell Transplantation Research Center, Research Institute for Oncology, Hematology and Cell Therapy, Tehran University of Medical Sciences, Tehran, Iran; 3 Hematology, Oncology and Stem Cell Transplantation Research Center, Research Institute for Oncology, Hematology and Cell Therapy, Tehran University of Medical Sciences, Tehran, Iran

**Keywords:** Acute myeloid leukemia, Measurable residual disease, Flow cytometry, Leukemic stem cells, Hematopoietic stem cells, CD45RA

## Abstract

**Background:**

Leukemic stem cells (LSCs) are the cellular reservoir most strongly implicated in relapse of acute myeloid leukemia, yet their operational detection by multiparameter flow cytometry remains challenging because of immunophenotypic overlap with normal progenitors and variability across assays. Including CD45RA in the CD34⁺CD38⁻ gating strategy substantially improves discrimination between malignant and normal stem/progenitor populations and thus enables more precise LSC enumeration in a single-tube format. Given the clinical importance of accurately quantifying the LSC compartment, we evaluated a refined single-tube flow cytometry assay that incorporates CD45RA within the CD34 + CD38- gate to increase specificity for the leukemic stem compartment.

**Methods:**

In a retrospective cohort of 109 AML bone marrow samples, measurable residual disease (MRD) was assessed with a conventional three-tube, 8-color panel and LSCs were enumerated using an adapted single-tube, 8-color panel defining LSCs as CD34 + CD38-CD45RA + . To ensure analytical reliability we applied a formal lower limit of quantification (LLOQ), defined empirically as a cluster of 50 CD45 + events; samples below the sample-specific LLOQ were not called positive. Positivity thresholds were set at ≥ 0.1% for MRD and ≥ 0.004% for LSCs. Group comparisons used the Mann–Whitney U test and associations were quantified by Pearson correlation.

**Results:**

LSCs were detectable in 28/109 (25.7%) patients, while MRD positivity was observed in 37/109 (33.9%) patients. A robust association was demonstrated between LSC presence and MRD positivity (p = 0.00035). The LSC burden was significantly elevated in MRD-positive patients, and concomitantly, MRD levels were profoundly higher in patients harboring detectable LSCs (p = 2.01 × 10⁻⁹). A strong positive correlation was observed between LSC and MRD levels across the entire cohort (R = 0.66, p = 3.2 × 10⁻^15^). LSC and MRD status were independent of sex, FLT3, or NPM1 mutation status. Immunophenotypic profiling of the LSC compartment revealed predominant aberrant co-expression of CD33 (89.3%) and a multi-marker cocktail (89.3%), with CD44 (67.9%) and CD123 (53.6%) also frequently observed.

**Conclusions:**

The implementation of a refined LSC detection assay, leveraging CD45RA gating and a stringent LLOQ, yields a specific and clinically actionable quantification of the LSC reservoir in AML. The strong correlation between the CD34 + CD38-CD45RA + LSC subset and MRD status suggests its potential as a complementary biomarker for residual disease monitoring; however, prospective validation in outcome-annotated cohorts is required to establish its prognostic utility and clinical applicability.

**Supplementary Information:**

The online version contains supplementary material available at 10.1186/s13287-026-05038-w.

## Background

Acute myeloid leukemia (AML) is a heterogeneous hematologic malignancy characterized by the rapid clonal expansion of aberrant myeloid precursors within the bone marrow that disrupts normal hematopoiesis [1]. Despite substantial advances in therapeutic modalities, from intensive cytotoxic regimens to an expanding armamentarium of targeted agents, relapse and treatment resistance remain major obstacles and contribute to persistently suboptimal long-term survival for many patients [2]. Relapse rates after initial complete remission are high, and relapsed or refractory AML is associated with poor overall survival and few durable responses to salvage therapy; thus, accurate detection of residual disease after treatment, termed measurable residual disease (MRD), has become a critical prognostic and clinical decision-making tool [3–6]. However, studies have shown that the 5-year estimated OS for the even MRD-negative group was below 70%. Multiple interrelated factors contribute to poor outcomes in AML, including adverse cytogenetic and molecular lesions, microenvironment-mediated protection, metabolic adaptations, and persistence of therapy-resistant cellular subpopulations, and among these, the persistence of leukemic stem cells (LSCs) is increasingly recognized as a central driver of relapse [7, 8].

Leukemic stem cells are a functionally defined subset of AML cells with the capacity for self-renewal, long-term repopulation, and hierarchical reconstitution of the leukemic clone; they are phenotypically and molecularly distinct from normal hematopoietic stem cells (HSCs) and are thought to seed relapse by surviving cytotoxic therapy and regenerating disease [7, 9, 10]. Work synthesizing LSC biology highlights their unique genetic/epigenetic programs, metabolic profiles, and protective interactions with the bone marrow niche, all of which underlie intrinsic and niche-mediated therapy resistance [11]. Quantification of the LSC compartment provides independent prognostic information, as higher LSC frequency and enriched stemness gene signatures are associated with increased relapse risk and poorer survival, and incorporating LSC assessment into conventional MRD evaluation improves risk stratification [12, 13]. Both retrospective and prospective studies have demonstrated that LSC frequency adds independent prognostic value [13–17]. The recent 2025 ELN-DAVID study led by Prof. Cloos and endorsed by ELN reported that LSC measurement achieves higher sensitivity than bulk-blast MRD, further supporting routine LSC monitoring [18]. Multiparameter flow cytometry (MFC) offers a practical method for identifying and enumerating these rare, aberrant immunophenotypes at single-cell resolution and for enabling serial MRD surveillance in clinical samples [19, 20].

Immunophenotypic studies show that LSCs are heterogeneous between patients and can be found in multiple cellular compartments, including CD34⁺CD38⁺, CD34⁺CD38⁻ and even CD34⁻ fractions, but the CD34⁺CD38⁻ compartment is consistently enriched for the most therapy-resistant and least immunogenic stem-like cells [21]. Functional assays such as aldehyde dehydrogenase activity and the side-population assay, together with molecular and cytogenetic confirmation, have validated that immunophenotypically defined CD34⁺CD38⁻ populations often contain bona fide LSC activity. LSCs commonly co-express a constellation of myeloid and aberrant markers, including CD13, CD33, CD123, CLEC12A (CLL-1), CD44, CD96, and TIM-3, as well as lineage-inappropriate antigens such as CD7 or CD56. These combined patterns allow discrimination from normal HSCs when analyzed in multiparameter panels. Recent practical advances in flow cytometry, including validated standardized 8-color panels and streamlined single-tube approaches informed by Zeijlemaker et al., enable sensitive detection of LSCs at low frequencies while reducing sample volume and assay complexity, thereby facilitating incorporation of LSC quantification into routine MRD workflows [20, 22].

Despite advances in standardized panels, the precise discrimination of leukemic stem cells (LSCs) from normal hematopoietic stem cells (HSCs) within the CD34⁺CD38⁻ compartment remains an analytical challenge, limiting the routine integration of LSC quantification into MRD workflows. We hypothesized that incorporating CD45RA, a marker absent on primitive HSCs but frequently re-expressed in AML, as a positive gating parameter would enhance the specificity of LSC identification. To test this, we implemented the Zeijlemaker-informed single-tube 8-color LSC panel, applying a refined gating strategy for CD34⁺CD38⁻CD45RA⁺ cells. The primary objectives of this study were to validate the reproducible assay for precise enumeration of the LSC population and to rigorously evaluate its association with MRD status in a cohort of AML patients.

## Methods

### Patient cohort and ethical considerations

This retrospective study consecutively screened patients with a confirmed diagnosis of acute myeloid leukemia (AML) referred for measurable residual disease (MRD) evaluation at the Research Institute for Oncology, Hematology and Cell Therapy, Tehran University of Medical Sciences, between 5 April 2025 and 22 September 2025. Bone marrow aspirates were excluded based on predefined quality criteria, including evidence of hemodilution, low viability, hemolysis, or clot formation. Furthermore, samples yielding fewer than 500,000 CD45⁺ leukocytes after processing were excluded from MRD analysis, and those with fewer than 1,250,000 CD45⁺ leukocytes were excluded from LSC analysis. These thresholds were established to ensure the reliability of multiparameter flow cytometry data acquisition and interpretation for rare cell populations.

The study protocol was approved by the Research Ethics Committee of the Research Institute for Oncology, Hematology and Cell Therapy, Tehran University of Medical Sciences (approval no. IR.TUMS.HORCSCT.REC.1403.024; 12 June 2024). To mitigate potential conflicts of interest, two study authors, Mohammad Vaezi (M.V.) and Ghasem Janbabaei (G.J.), who are members of this committee, were formally recused from all deliberations, voting, and decisions pertaining to the review and approval of this study. Ethical review and final approval were conducted solely by the remaining independent committee members in accordance with institutional guidelines. Written informed consent was obtained from all participants.

### Sample collection and processing

Bone marrow aspirates were collected in EDTA-containing tubes at diagnosis and during treatment follow-up. Samples were immediately transported to the flow cytometry laboratory. Sample processing, including red blood cell lysis, washing, and staining, was performed according to the procedural steps outlined in the EuroFlow bulk lysis standard operating procedure (SOP version 2.0, June 2025).

Briefly, red blood cell lysis was carried out using a 1X ammonium chloride solution (Pharm Lyse, BD Biosciences) for 15 min at room temperature. The resulting leukocyte pellet was washed twice with a filtered phosphate-buffered saline (PBS) solution, pH 7.4, supplemented with 0.2% bovine serum albumin (BSA), 2 mM EDTA, and 0.09% sodium azide (washing buffer). Following the final wash, cells were resuspended in washing buffer for subsequent antibody staining. As detailed in the previous section, only samples yielding a minimum of 500,000 CD45⁺ leukocytes after processing were used for MRD analysis, and those with at least 1,250,000 CD45⁺ leukocytes were used for LSC analysis.

### Flow cytometry antibody panel for MRD and LSC identification

The MRD antibody panel consisted of 3 tubes that utilize backbone markers (CD45, HLA-DR, CD34, CD117, CD13) for MRD detection in AML (Table 1). Fluorochromes were strategically allocated across all tubes to minimize spectral overlap and maximize resolution, ensuring compatibility with EuroFlow-compliant platforms.

**Table 1:** Multicolor flow cytometry antibody panel for differentiating measurable residual disease

	FITC	Clone	PE	Clone	PerCP-CY5.5	Clone	PE-CY7	Clone	APC	Clone	APC-H7	Clone	BV421	Clone	V500	Clone
Tube 1	CD7	124-1D1	CD56	MEM-188	CD34	581	CD117	104D2	CD33	WM53	HLA-DR	L243	CD13	WM15	CD45	2D1
Tube 2	CD15	MMA	CD22	S-HCL-1	CD34	581	CD117	104D2	CD19	LT19	HLA-DR	L243	CD13	WM15	CD45	2D1
Tube 3	CD36	CB38	CD14	MEM-15	CD34	581	CD117	104D2	CD64	10.1	HLA-DR	L243	CD13	WM15	CD45	2D1

Leukemic stem cells (LSCs) were identified using an 8-color, 13-antibody flow cytometry panel adapted from Zeijlemaker et al. (2019) (Table 2).

**Table 2:** Multicolor flow cytometry antibody panel for differentiating leukemic stem cells

FITC	Clone	PE	Clone	PerCP-CY5.5	Clone	PE-CY7	Clone	APC	Clone	APC-H7	Clone	BV421	Clone	V500	Clone
CD45RA	MEM-56	Clec12a	50C1	CD123	6H6	CD34	8G12	CD38	HIT2	CD44	MEM-85	CD33	WM53	CD45	2D1
		TIM-3	7D3												
		CD7	124-1D1												
		CD11b	MEM-174												
		CD22	S-HCL-1												
		CD56	MEM-188												

### Data acquisition

Flow cytometric data were acquired on a BD FACSLyric flow cytometer (BD Biosciences) calibrated daily using CS&T beads. A minimum of 0.5 × 10⁶ and 1.25 × 10⁶ events per sample were recorded to ensure sensitivity for rare MRD and LSC populations, respectively.

### Gating strategy and identification of MRD and LSCs

**Fig. 1:**
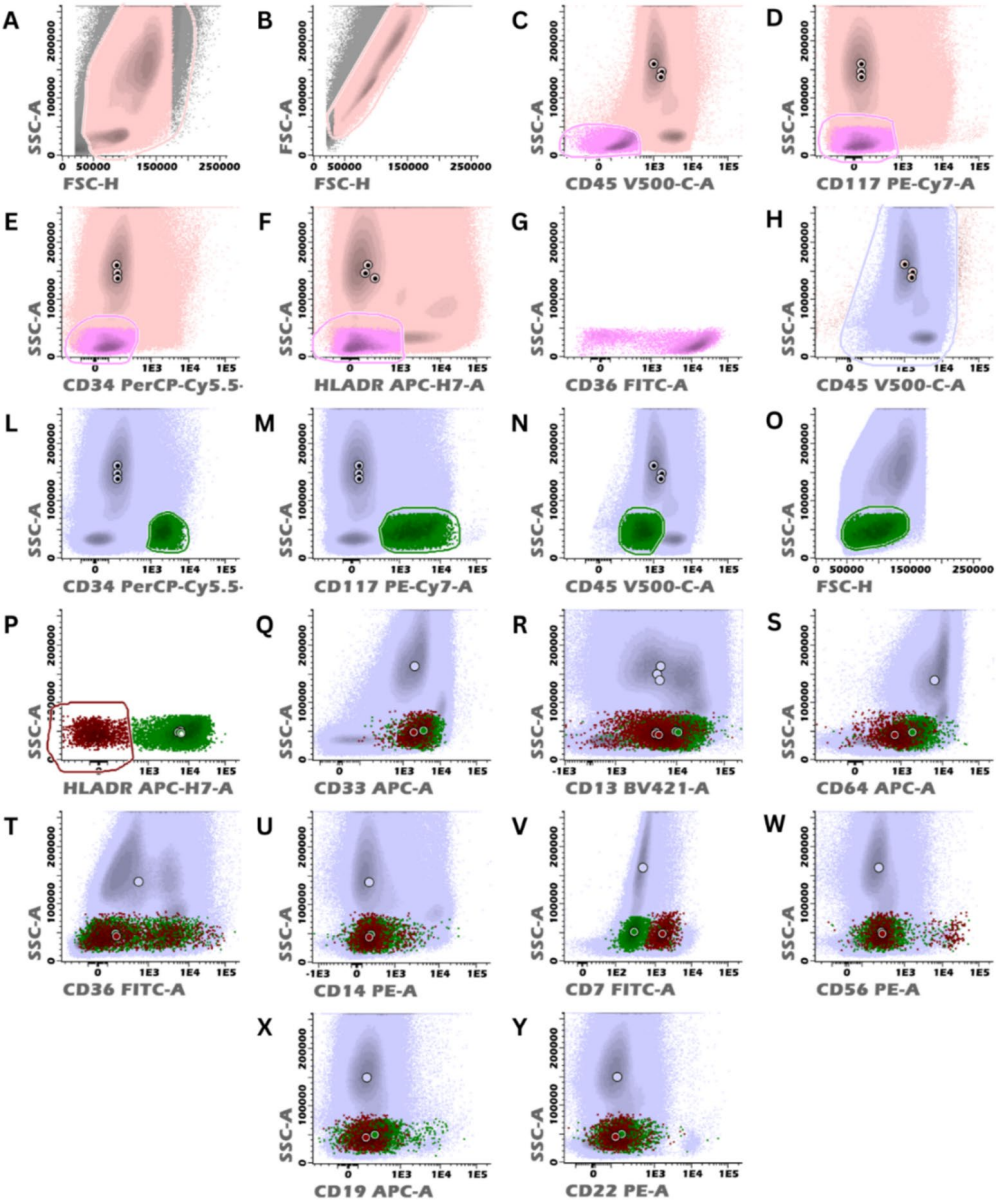
Flow cytometric gating strategy for measurable residual disease (MRD) detection in acute myeloid leukemia. Sequential plots demonstrate the analytical pipeline: **A-B** exclusion of debris and doublets; **C-G** gating to exclude erythroid cells based on negative expression of CD45, CD34, CD117, CD13, and HLA-DR; **H** identification of leukocytes (CD45 +); **L-O** sequential identification of myeloblast populations (CD34 + CD117 + , CD34-CD117 + , CD34 + CD117-, CD34-CD117-) based on backbone markers; **P-Y** subsequent immunophenotypic characterization for leukemia-associated immunophenotype (LAIP) identification using the full antibody panel

Data analysis was conducted using Infinicyt software (Cytognos, Salamanca, Spain, V 2.0.6), which employs advanced multidimensional visualization and clustering algorithms for high-resolution gating [23]. The gating strategy was implemented with exacting precision and comprised several sequential steps (Fig. 1). Initially, debris, non-viable cells, and doublets were excluded using forward and side scatter parameters (FSC-A/FSC-H and SSC-A/FSC-H). Erythroid cells were excluded based on their negative expression of CD45, CD34, CD117, CD13, and HLA-DR, and subsequently, leukocytes were identified by plotting side scatter (SSC) against CD45.

For MRD assessment, leukocyte events were further analyzed. Normal myeloblasts were defined based on the co-expression of CD34, CD117, CD13, and HLA-DR, along with moderate CD45 expression. MRD-positive populations were identified using a leukemia-associated immunophenotype (LAIP) and difference from normal (DfN) approach (LAIP-based DFN method). This involved the independent evaluation of CD34⁺CD117⁺, CD34⁻CD117⁺, CD34⁺CD117⁻, and CD34⁻CD117⁻ populations, followed by detailed immunophenotypic characterization using the additional markers in the panel. Figure 1 shows the gating strategy for detecting MRD.

**Fig. 2:**
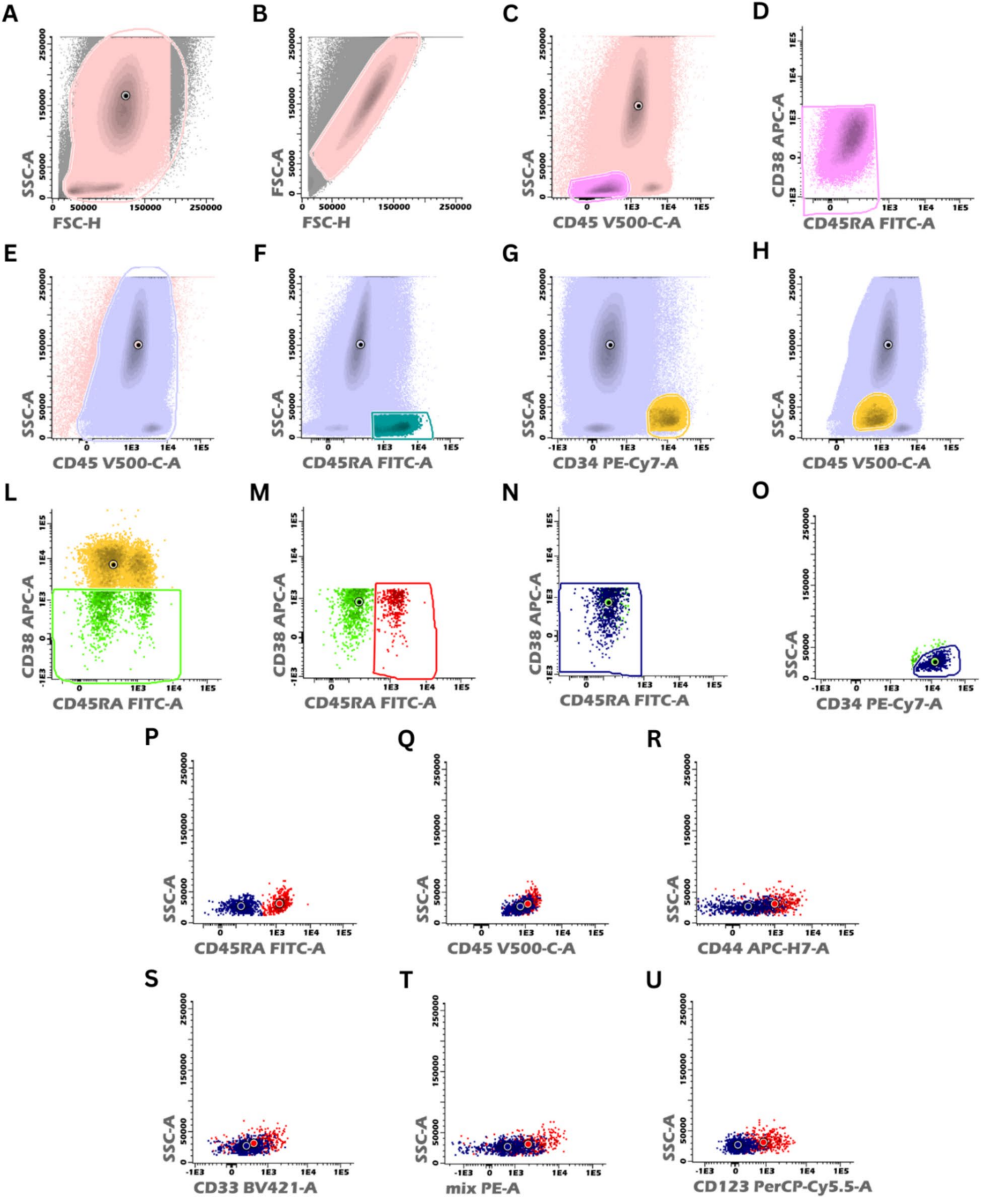
Gating strategy for the identification of leukemic stem cells (LSCs) and hematopoietic stem cells (HSCs). The methodology for delineating the CD34 + CD38- stem cell compartment is shown: **A–B** exclusion of debris and doublets; **C–D** exclusion of erythroid cells (CD45-CD45RA-CD38-); **E** identification of leukocytes; **F** use of CD44 + CD33- lymphocytes as an internal control to set the CD45RA positivity threshold; **G–H** identification of CD34 + progenitor cells; **L** isolation of the CD34 + CD38- stem cell pool; **M** definitive identification of LSCs as CD34 + CD38-CD45RA + cells; **N** identification of HSCs as CD34 + CD38-CD45RA- cells; **P–U** comparative immunophenotypic analysis of aberrant marker expression (e.g., CD123, CD33, CD44) on the defined LSC and HSC populations

For LSC identification, a distinct gating strategy was applied. LSCs and HSCs were defined as CD34⁺CD38⁻CD45RA⁺ and CD34⁺CD38⁻CD45RA^−^ cells, respectively. Establishing the CD38-negative gate required careful consideration, given the lack of a universal cutoff. Among the methods proposed by Cloos et al. (2018), the approach utilizing the upper border of the red cell fraction to define the CD38-negative threshold was selected for this study [24]. This method was chosen because it does not require specialized beads and is more appropriate for personalized sample analysis, reducing the risk of false cutoffs that can occur with fixed median fluorescence intensity (MFI) thresholds in intra-run analyses. Moreover, the positive cutoff for CD45RA expression was defined using an internal control. CD44⁺CD33⁻CD45⁺ cells within the lymphocyte gate were selected, and a distinct population gap was observed in the CD45RA plot for these cells. This gap was used to establish the threshold for defining CD45RA positivity. For samples in which this gap was not observed, the higher red cell fraction for CD45RA was used as the cut-off for CD45RA (Fig. 2).

### Limit of detection and limit of quantification

The limit of detection (LOD) was established at 20 CD45^+^ events exhibiting a suspected leukemic phenotype, while the lower limit of quantification (LLOQ) was defined as 50 CD45^+^ events, in accordance with European LeukemiaNet (ELN) guidelines [25]. We calculated the LLOQ for each sample, and only the samples that had a population above the LLOQ and the chosen cut-offs (≥ 0.004% and ≥ 0.1%) were assigned as positive MFC-LSC and MFC-MRD, respectively.

### Statistical analysis

Statistical analyses were conducted in R (v4.3.1). Continuous variables, including LSC frequency and myeloblast percentage, are reported as median (interquartile range) unless stated otherwise. Associations between continuous MRD values and cell-subset frequencies (expressed as percentage of CD45 + events) were evaluated primarily with Pearson correlation coefficients. Two compartments were predefined for these analyses: the broad CD34 + CD38- pool (which may include both normal HSCs and malignant LSCs) and the restricted CD34 + CD38-CD45RA + LSC subset. Bivariate normality was assessed visually and with the Shapiro–Wilk test; when normality assumptions were not met, Spearman rank correlations were computed as a sensitivity analysis. Group comparisons for non-normally distributed data used the Mann–Whitney U test. Two-tailed p-values < 0.05 were considered statistically significant.

## Results

### Patient characteristics

A total of 109 patients with newly diagnosed acute myeloid leukemia were included. Age was available for 102 patients, with a median of 42.5 years (range 6–74) and a mean of 42.48 years. The cohort comprised 57 female and 52 male patients.

### Flow cytometry acquisition and CD34 expression

Cytometric acquisition confirmed deep sampling across specimens. Total recorded events per sample were 1.25 × 10⁶ to 5.4 × 10⁶ (mean ≈ 3.46 × 10⁶). After exclusion of CD45-negative events, leukocyte event counts were 1.4 × 10⁶ to 4.8 × 10⁶ (mean ≈ 2.8 × 10⁶). CD34 expression was highly skewed, with a median of 0.7097 percent, a mean of 8.8941 percent, and an overall range of 0.0118 to 96.7213 percent.

### Distribution of MRD and LSCs

**Fig. 3:**
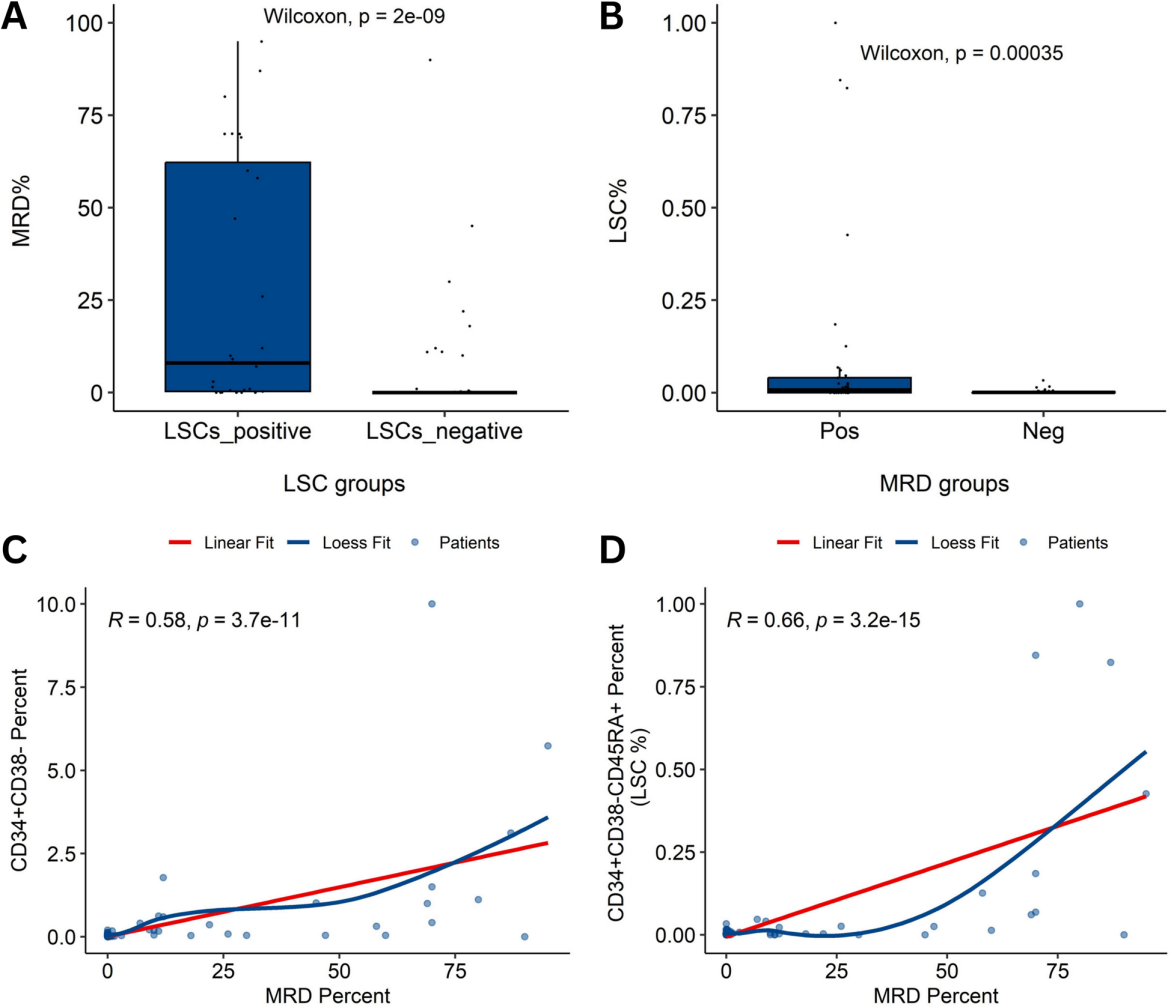
Association between measurable residual disease (MRD) and leukemic stem cell (LSC) burden. **A** MRD levels were significantly elevated in patients with detectable LSCs compared to those without. **B** Conversely, the LSC frequency was considerably higher in MRD-positive patients than in MRD-negative patients. **C** Scatter plot showing a modest positive correlation between the percentage of CD34⁺CD38⁻ cells and MRD levels across individual patients. **D** scatter plot showing a stronger positive correlation between the percentage of CD34⁺CD38⁻CD45RA⁺ LSCs and MRD levels across individual patients. Statistical significance in A and B was determined by the Mann–Whitney U test, and in C and D by Pearson correlation; ***p < 0.001

Overall, 37 of 109 samples (33.9%) were MRD-positive and 28 of 109 samples (25.7%) were LSC-positive. Cross-classification produced four groups: MRD − /LSC − , 66 patients (60.6%); MRD + /LSC − , 15 patients (13.8%); MRD − /LSC + , 6 patients (5.5%); and MRD + /LSC + , 22 patients (20.2%). The MRD burden was substantially higher among patients with detectable LSCs compared with those without (p = 2.01 × 10^–9^) (Fig. 3A). Conversely, the fraction of LSCs was significantly greater in MRD-positive patients compared with MRD-negative patients (p = 0.00035) (Fig. 3B).

In the cohort (n = 109), the broad CD34 + CD38- pool showed a moderate linear association with MRD (R = 0.58, p = 3.7 × 10^–11^; Fig. 3C). The more restricted CD34 + CD38-CD45RA + subset correlated more strongly with MRD (R = 0.66, p = 3.2 × 10^–15^; Fig. 3D).

### Association with age and other clinical variables

**Fig. 4:**
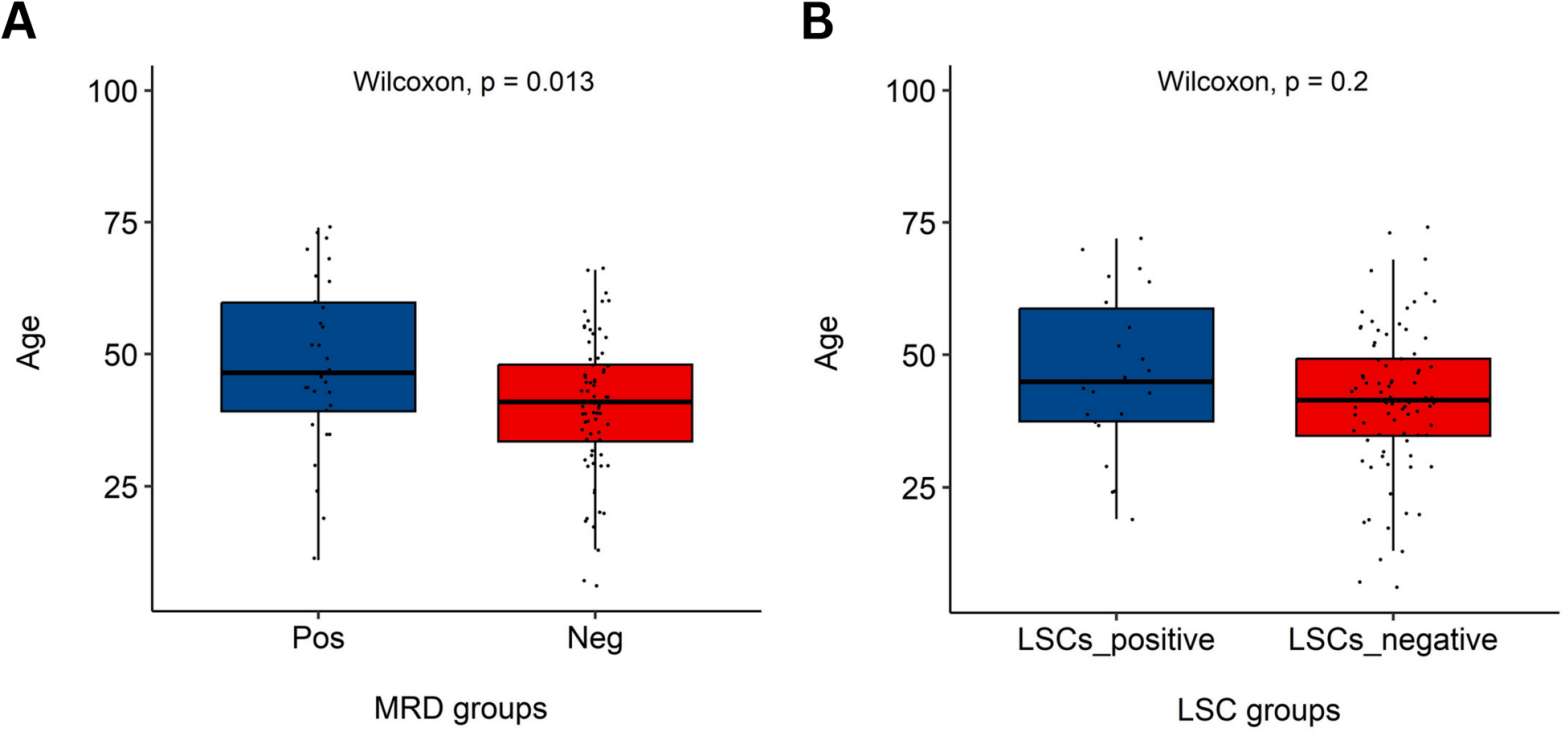
Relationship between patient age and disease status. **A** MRD-positive patients were significantly older than MRD-negative patients. **B** In contrast, no statistically significant difference in age was observed between LSC-positive and LSC-negative patient groups. The central line in the box plots represents the median, the box extends from the 25th to the 75th percentile, and whiskers show the range. P-values were calculated using the Mann–Whitney U test

Age was higher among MRD-positive patients, with a mean of 48.3 years (SD 16.1, n = 37) for MRD-positive and 40.0 years (SD 13.0, n = 72) for MRD-negative (p = 0.013) (Table 3 and Fig. 4A). Age was also higher in LSC-positive patients compared with LSC-negative patients; however, this difference did not reach statistical significance (p = 0.20) (Table 3 and Fig. 4B). No significant associations were observed between MRD status or LSC status and sex, FLT3 mutation status, or NPM1 mutation status, as assessed by chi-square or Fisher’s exact tests where appropriate.

**Table 3:** Patient characteristics stratified by measurable residual disease (mrd) and leukemic stem cell (LSC) status

Characteristic	MRD negative (n = 72)	MRD positive (n = 37)	p-value	LSCs negative (n = 81)	LSCs positive (n = 28)	p-value
Continuous variables, Mean ± SD						
% LSCs	0.002 ± 0.005	0.103 ± 0.250	< 0.001	—	—	—
% MRD	—	—	—	3.10 ± 12.0	27.8 ± 33.9	0.0004
Age (years)	40.0 ± 13.0	48.3 ± 16.1	0.013	41.4 ± 14.1	46.5 ± 15.3	0.200
Categorical variables, n (%)						
Sex						
Female	39 (54.2%)	18 (48.6%)	0.686	42 (51.9%)	15 (53.6%)	1.000
Male	33 (45.8%)	19 (51.4%)		39 (48.1%)	13 (46.4%)	
FLT3 status						
Negative	27 (73.0%)	11 (68.8%)	0.751	30 (75.0%)	8 (61.5%)	0.480
Positive	10 (27.0%)	5 (31.2%)		10 (25.0%)	5 (38.5%)	
NPM1 status						
Negative	29 (80.6%)	12 (85.7%)	1.000	30 (81.1%)	11 (84.6%)	1.000
Positive	7 (19.4%)	2 (14.3%)		7 (18.9%)	2 (15.4%)

### Aberrant immunophenotypic features in MRD and LSC analyses

A detailed analysis was conducted to characterize and compare the immunophenotypic landscapes of the bulk MRD population and the refined LSC compartment, with further stratification by co-detection status.

**Fig. 5:**
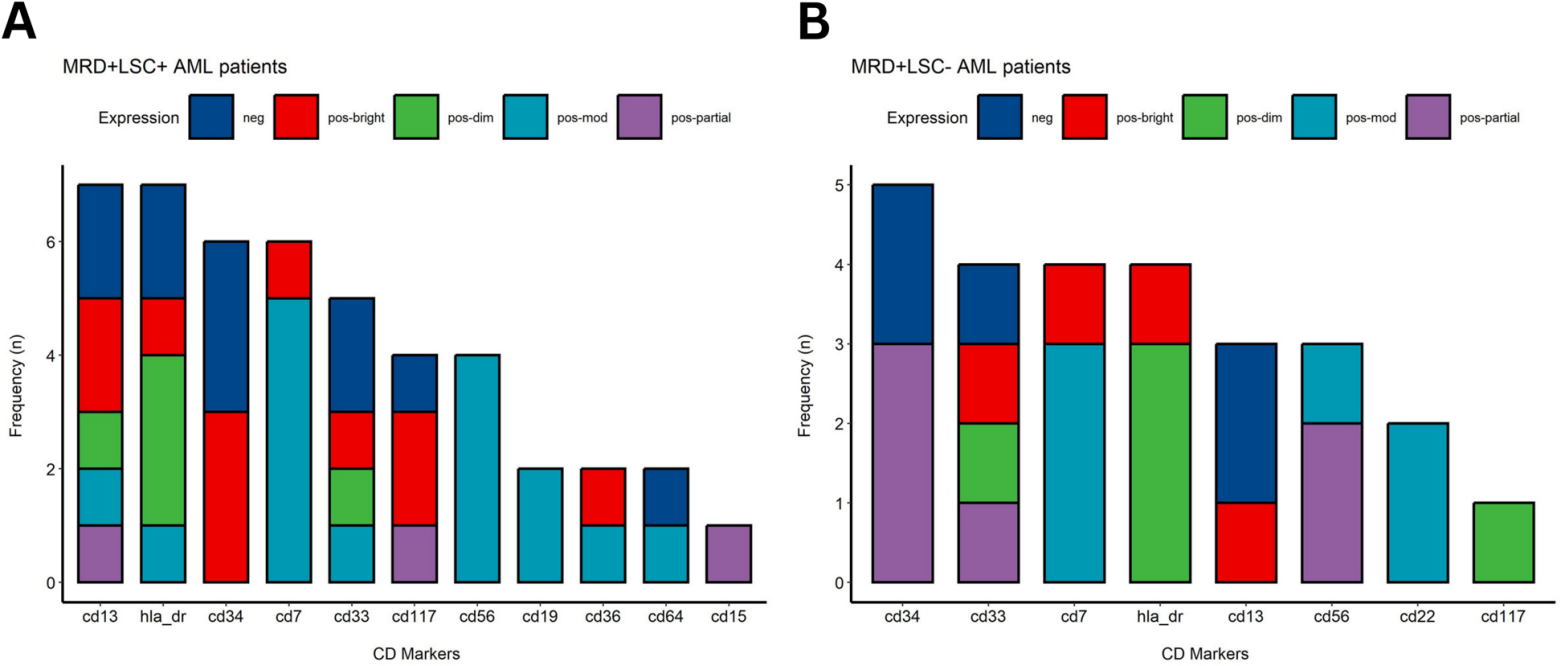
Spectrum of immunophenotypic aberrancies in MRD-positive populations. The frequency of aberrant marker expression is shown for MRD-positive patients who were also **A** LSC-positive (MRD + LSC +) and **B** LSC-negative (MRD + LSC-). The data illustrate the heterogeneity of leukemia-associated immunophenotypes (LAIPs) detected within the MRD compartment

Analysis of the MRD compartment across all 37 MRD-positive patients (summarized in Supplementary Table S1) revealed a heterogeneous pattern of leukemia-associated immunophenotypes (LAIPs). The most frequent aberrancies involved markers of lineage infidelity, with aberrant CD7 expression being the most prevalent, identified in 10 samples (27.0%). This was followed by aberrant CD56 expression in 7 samples (18.9%). Asynchronous or heterogeneous expression of core myeloid markers was also common, observed in CD34, CD13, and CD33 (each in 6 samples, 16.2%). Stratification of these MRD-positive cases by LSC status revealed distinct immunophenotypic patterns (Fig. 5). In the MRD + LSC + subgroup, the residual blast population displayed a broader spectrum of aberrancies. Notably, a high frequency of HLA-DR expression was observed, alongside frequent asynchronous patterns in CD34 and CD13. In contrast, the MRD + LSC- subgroup exhibited a more restricted aberrancy profile, with a lower overall frequency of most markers but a notable presence of aberrancies in CD34, CD33, and CD7.

**Fig. 6:**
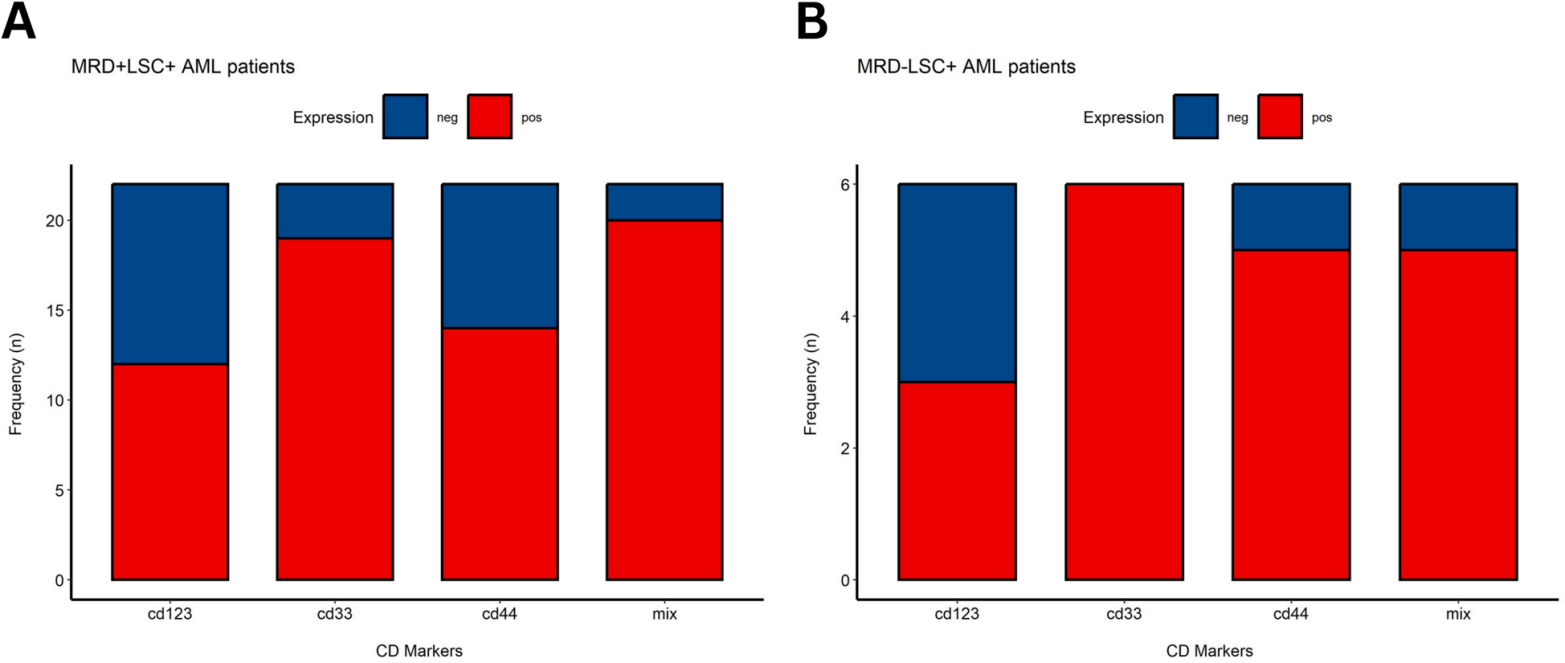
Immunophenotypic profile of the leukemic stem cell (LSC) compartment. The bar chart depicts the frequency of canonical LSC-associated marker expression (CD123, CD33, CD44, and a mixed marker cocktail) on CD34 + CD38-CD45RA + LSCs, stratified by the MRD status of the patient. This analysis demonstrates the high prevalence of these aberrant markers within the defined LSC population

Within the rigorously defined LSC compartment (CD34⁺CD38⁻CD45RA⁺), a highly consistent immunophenotypic signature was observed across the 28 LSC-positive patients (Supplementary Table S2). The vast majority of samples exhibited co-expression of CD33 (89.3%) and the mixed marker cocktail (89.3%), underscoring their role as cornerstone markers for LSC identification. CD44 and CD123 were also frequently expressed, present in 67.9 and 53.6% of LSC-positive samples, respectively. Further stratification of LSC-positive patients by MRD status demonstrated that this core immunophenotype was remarkably stable (Fig. 6). The expression frequencies of CD123, CD33, CD44, and the mixed cocktail were consistently high in both the LSC + MRD + and LSC + MRD- subgroups.

## Discussion

This study robustly demonstrates a significant association between the presence of CD34⁺CD38⁻CD45RA⁺ LSCs and MRD status in AML patients. By employing the standardized single-tube assay pioneered by Zeijlemaker et al. and refining the conventional LSC gate with the inclusion of CD45RA as a positive selector, we provide a biologically and technically enhanced window into the primitive leukemic compartment implicated in disease persistence. Our findings, contextualized within the evolving landscape of LSC biology, validate the compartmentalization of relapse risk within a phenotypically defined stem-cell pool and underscore its potential for refining post-therapy risk stratification.

Our operational definition of LSCs as CD34⁺CD38⁻CD45RA⁺ cells is a deliberate refinement built upon the foundational work of Zeijlemaker et al., who designed a comprehensive single-tube 8-color assay for practical LSC quantification [20]. Their systematic evaluation of 13 markers established that a cocktail of six antibodies (CLL-1, TIM-3, CD7, CD11b, CD22, CD56) in the PE channel, combined with single stains for CD44, CD33, and CD123, could capture the vast heterogeneity of LSCs while conserving precious bone marrow material. Our study explicitly leverages CD45RA, a key component of their tube, not just as an ancillary marker but as a critical, positive gating parameter to discriminate LSCs from normal hematopoietic stem cells (HSCs) [24].

This refinement is grounded in the developmental biology of hematopoiesis. CD45RA is a marker associated with lymphoid-primed multipotent progenitors (LMPPs), a lineage-committed stage downstream of pluripotent HSCs [26]. Its aberrant retention or re-expression on AML stem cells is a recognized feature of leukemogenesis, helping to distinguish them from the CD45RA-negative phenotype of true, multipotent normal HSCs [26, 27]. This approach directly addresses a key analytical challenge highlighted in subsequent validations, such as the prospective HOVON-SAKK132 trial, which used a nearly identical tube [16]. While that trial confirmed the prognostic power of the CD34⁺CD38⁻ compartment, it also underscored the challenges of rare-event analysis, particularly the potential for false positives or overestimation when applying an ultra-sensitive “zero-event cutoff post-chemotherapy [16]. Our use of CD45RA as a positive selector aims to enrich for a population with a higher probability of leukemic “stemness”, thereby increasing the assay's clinical specificity by more effectively excluding residual normal HSCs that may persist after therapy.

A notable finding in our cohort was the lack of a significant association between MRD/LSC status and the presence of FLT3-ITD or NPM1 mutations. This may initially seem to conflict with studies linking adverse genetics to poor outcomes. However, this discrepancy is resolved when considering the phenotypic definitions of disease subsets. Recent studies reinforce that CD34-negative AML, an entity highly enriched for NPM1 mutations, represents a distinct biological and clinical entity with a more favorable prognosis [28, 29]. Our CD45RA + LSC definition, by virtue of being nested within the CD34 + compartment, is inherently selective for LSCs in CD34-positive disease. This likely explains the lack of association with NPM1, which is often (though not exclusively) associated with CD34-negative leukemias. Furthermore, the prospective HOVON-SAKK132 data showed that LSC burden provides prognostic information independent of ELN risk stratification [16]. This suggests that the biological aggressiveness captured by LSC measurement, potentially driven by a stem-cell transcriptional program and self-renewal capacity, can cut across traditional genetic categories. Our results align with this concept, indicating that the persistence of a CD45RA + LSC reservoir post-therapy is a poor prognostic sign, irrespective of the FLT3 or NPM1 mutation status at diagnosis. This supports the use of LSC monitoring as an orthogonal, functional risk assessment tool that complements, rather than simply mirrors, static genetic data obtained at diagnosis.

The sensitivity and reliability of flow cytometric LSC detection are intrinsically linked to various key technical parameters, including the total number of events acquired and the statistical thresholds used to define positivity [18, 24, 30]. Our study introduces a critical methodological refinement by formally calculating and applying a Lower Limit of Quantification (LLOQ) for each sample. We defined the LLOQ empirically as a cluster of 50 events within the acquired leukocyte population, ensuring that only populations above this statistically defined threshold of reliable detection are classified as positive. This approach, using established cut-offs of ≥ 0.004% for MFC-LSC and ≥ 0.1% for MFC-MRD, provides a robust guard against the false-positive assignment that can occur from analyzing minor, potentially nonspecific events in the background noise of flow cytometry data [24]. This methodology stands in direct contrast to the foundational Zeijlemaker et al. work, which used a much lower threshold of a cluster containing more than 5 cells to identify LSCs [20]. While their highly sensitive definition is crucial for discovering and characterizing rare populations in a research context, our application of a higher, statistically grounded event threshold (50 events) enhances reproducibility and clinical specificity for outcome prediction, as it requires a more substantial and confident cluster of cells to call a sample positive. While this rigorous standard enhances the clinical specificity of our results, we acknowledge its inherent trade-off: the potential to miss genuine minor LSC populations that fall below the LLOQ, which may still have clinical significance.

Our implementation of a formal LLOQ places our work in the context of an evolving field still grappling with standardization. Earlier studies, such as Kamel et al., used relatively low event counts (50,000 CD45 + events) and cohort-based median cut-offs, which are practical but may lack sensitivity and universal applicability [31]. The pioneering work of the Zeijlemaker/HOVON group has been instrumental in advancing the field toward deep sampling, with their protocol recommending acquiring 4 million WBCs to reliably detect these rare cells [25]. Their subsequent clinical validations have employed a well-defined diagnostic cut-off (≥ 0.03% of WBCs) and an ultra-sensitive, albeit challenging, post-induction threshold of “any detectable cluster” (> 0.00000%). While maximally sensitive, this “zero-tolerance” approach, especially when paired with a low event-cluster definition (≥ 5 cells), requires immense technical expertise to avoid false positives from background staining or regenerating normal progenitors. In contrast, the Reuvekamp et al. (2025) study demonstrated the importance of context-specific cut-offs, deriving distinct thresholds for HMA-treated patients (0.01% at diagnosis, 0.001% post-therapy). Our study bridges these concepts by combining the principle of deep event acquisition with a statistically grounded LLOQ based on a 50-event cluster. This provides a reproducible framework that balances the high sensitivity of the Zeijlemaker assay with enhanced specificity, making it a more robust and defensible tool for multi-institutional clinical trials and eventual routine clinical practice by providing greater confidence in positive calls.

Our approach is subject to several important limitations. First, our stringent application of an LLOQ, while crucial for ensuring specificity and minimizing false positives, carries the inherent risk of missing genuine minor LSC populations that fall below this statistical threshold. Such sub-LLOQ populations may still possess clinical significance and contribute to late relapse, representing a potential trade-off between analytical reliability and maximum sensitivity. Second, and more critically, our assay is confined to the CD34-positive compartment, which is a significant analytical gap, as it is well-established that leukemia-initiating capacity can also reside in CD34-negative blasts. While CD34 + CD38 − cells are indeed considered the most therapy-resistant and least immunogenic population in vitro and in vivo, the biological and clinical relevance of CD34-negative LSCs, particularly in genetic subtypes like NPM1-mutated AML or in the context of novel therapies like venetoclax, cannot be ignored [32–34]. The work of Reuvekamp et al. conclusively showed that markers effective in the CD34 + compartment, like CD133, are not suitable surrogates in CD34-negative disease [28]. Finally, outcome evaluation was not an objective of this assay-validation study; accordingly, we did not assess the prognostic value of the four MRD/LSC groups to demonstrate the superior prognostic performance of the CD45RA-enriched gating strategy. The study was designed to establish analytical reproducibility and a statistically justified LLOQ for rare-event detection rather than to test clinical endpoints. Given existing evidence that LSC quantification can carry prognostic information and recent expert recommendations to further evaluate LSCs within MRD workflows, omission of survival analyses here does not diminish the technical contribution of the work; it preserves a clear scope and avoids underpowered or potentially misleading prognostic inferences.

To address these gaps, future studies must therefore pursue a dual path. Firstly, there is an urgent need for the field to harmonize and standardize the entire LSC detection workflow. This includes defining consensus time points for assessment, minimum event acquisition requirements, statistically justified LLOQs, validated clinical cut-offs, unified gating strategies, and, ultimately, a standardized antibody panel. Such multi-institutional efforts are paramount for generating comparable data across clinical trials. Secondly, building upon the foundational “single-tube” concept, future assay iterations must incorporate parallel gating strategies to comprehensively capture both CD34-positive and CD34-negative LSC compartments. As suggested, this may involve leveraging markers like CD117, CD33, or other primitive antigens as a backbone for the CD34-negative fraction [28, 35, 36]. Finally, pooled or multicenter prospective cohorts with pre-specified endpoints and sufficient numbers of discordant cases (MRD − /LSC +) are required to evaluate prognostic impact. Only by addressing these analytical and study-design challenges can LSC assessment be advanced toward robust, generalizable clinical application.

## Conclusion

Using a Zeijlemaker-informed single-tube panel with CD45RA gating, we demonstrate a reproducible workflow for enumerating a CD34 + CD38 − CD45RA + leukemic stem cell population that shows strong concordance with bulk MRD. The workflow enforces a statistically justified LLOQ to reduce false positives and improve inter-sample comparability, acknowledging the resulting trade-off in sensitivity for events below that threshold. The approach reduces the likelihood of contamination from normal HSCs and strengthens methodological rigor for rare-event detection. However, assessing the independent prognostic impact and integrating it into risk-stratification algorithms will require larger, prospectively followed cohorts or pooled multicenter analyses with sufficient representation of discordant MRD − /LSC + cases. We therefore recommend follow-up studies specifically powered to evaluate whether CD45RA-defined LSC quantification adds prognostic value beyond standard MRD testing.

## Supplementary Information

Supplementary Material 1.

## Data Availability

The datasets analyzed during the current study are available from the corresponding author on reasonable request.

## Abbreviations

AML: Acute myeloid leukemia
APC: Allophycocyanin
BV: Brilliant violet
CLL-1: C-Type lectin domain family 12 member A
DFN: Difference from normal
EDTA: Ethylenediaminetetraacetic acid
ELN: European leukemianet
FBS: Fetal bovine serum
FITC: Fluorescein isothiocyanate
FLT3: FMS-like tyrosine kinase 3
FSC: Forward scatter
HMA: Hypomethylating agent
HSC: Hematopoietic stem cell
HLA-DR: Human leukocyte antigen–DR isotype
ITD: Internal tandem duplication
LAIP: Leukemia-associated immunophenotype
LLOQ: Lower limit of quantification
LOD: Limit of detection
LMPP: Lymphoid-primed multipotent progenitor
LSC: Leukemic stem cell
MFC: Multiparameter flow cytometry
MFI: Median fluorescence intensity
MNC: Mononuclear cell
MRD: Measurable residual disease
NPM1: Nucleophosmin 1
PBS: Phosphate-buffered saline
PE: Phycoerythrin
PerCP: Peridinin-chlorophyll-protein
SSC: Side scatter
TIM-3: T-cell immunoglobulin and mucin-domain containing-3
WHO: World Health Organization
