# Supplementary material for: Refined detection of CD34⁺CD38⁻CD45RA⁺ leukemic stem cells using a single-tube flow cytometry assay and its strong association with measurable residual disease in acute myeloid leukemia: a retrospective cohort study
Source: Stem Cell Res Ther. 2026 May 2;17:229. doi: 10.1186/s13287-026-05038-w (PMC13312572; doi:10.1186/s13287-026-05038-w)
Supplement: Supplementary file 1 — Supplementary Material 1. [file 13287_2026_5038_MOESM1_ESM.docx]

**Supplementary Table**

**Supplementary Table 1. MRD aberrancies**

| CD Marker | Total | Expression | Count | Frequency |
| --- | --- | --- | --- | --- |
| CD34 | 11 | neg | 5 | 13.51351 |
|  |  | pos-bright | 3 | 8.108108 |
|  |  | pos-partial | 3 | 8.108108 |
| CD117 | 5 | pos-bright | 2 | 5.405405 |
|  |  | neg | 1 | 2.702703 |
|  |  | pos-dim | 1 | 2.702703 |
|  |  | pos-partial | 1 | 2.702703 |
| HLA-DR | 10 | pos-dim | 6 | 16.21622 |
|  |  | neg | 2 | 5.405405 |
|  |  | pos-bright | 2 | 5.405405 |
| CD13 | 9 | neg | 4 | 10.81081 |
|  |  | pos-bright | 3 | 8.108108 |
|  |  | pos-dim | 1 | 2.702703 |
|  |  | pos-partial | 1 | 2.702703 |
| CD33 | 8 | neg | 3 | 8.108108 |
|  |  | pos-bright | 2 | 5.405405 |
|  |  | pos-dim | 2 | 5.405405 |
|  |  | pos-partial | 1 | 2.702703 |
| CD7 | 10 | pos-mod | 8 | 21.62162 |
|  |  | pos-bright | 2 | 5.405405 |
| CD56 | 5 | pos-mod | 5 | 13.51351 |
| CD15 | 1 | pos-partial | 1 | 2.702703 |
| CD19 | 2 | pos-mod | 2 | 5.405405 |
| CD22 | 2 | pos-mod | 2 | 5.405405 |
| CD36 | 2 | pos-bright | 1 | 2.702703 |
|  |  | pos-mod | 1 | 2.702703 |
| CD56 | 2 | pos-partial | 2 | 5.405405 |
| CD64 | 2 | neg | 1 | 2.702703 |
|  |  | pos-mod | 1 | 2.702703 |

**Supplementary Table 2. LSCs immunophenotype**

| CD Marker | Expression | Count | Total | Frequency |
| --- | --- | --- | --- | --- |
| cd123 | pos | 15 | 28 | 53.57143 |
|  | neg | 13 | 28 | 46.42857 |
| cd33 | pos | 25 | 28 | 89.28571 |
|  | neg | 3 | 28 | 10.71429 |
| cd44 | pos | 19 | 28 | 67.85714 |
|  | neg | 9 | 28 | 32.14286 |
| mix | pos | 25 | 28 | 89.28571 |
|  | neg | 3 | 28 | 10.71429 |
